# Hexon Modification to Improve the Activity of Oncolytic Adenovirus Vectors against Neoplastic and Stromal Cells in Pancreatic Cancer

**DOI:** 10.1371/journal.pone.0117254

**Published:** 2015-02-18

**Authors:** Tanja Lucas, Karim Benihoud, Frédéric Vigant, Christoph Q. Andreas Schmidt, Max G. Bachem, Thomas Simmet, Stefan Kochanek

**Affiliations:** 1 Department of Gene Therapy, Ulm University, Ulm, Germany; 2 Univ. Paris-Sud, Orsay Cedex, France and CNRS UMR 8203, Institut Gustave Roussy, Villejuif Cedex, France; 3 Institute of Pharmacology of Natural Products & Clinical Pharmacology, Ulm University, Ulm, Germany; 4 Tierforschungszentrum, Ulm University, Ulm, Germany; 5 Department of Clinical Chemistry, Ulm University, Ulm, Germany; French National Centre for Scientific Research, FRANCE

## Abstract

Primary pancreatic carcinoma has an unfavourable prognosis and standard treatment strategies mostly fail in advanced cases. Virotherapy might overcome this resistance to current treatment modalities. However, data from clinical studies with oncolytic viruses, including replicating adenoviral (Ad) vectors, have shown only limited activity against pancreatic cancer and other carcinomas. Since pancreatic carcinomas have a complex tumor architecture and frequently a strong stromal compartment consisting of non-neoplastic cell types (mainly pancreatic stellate cells = hPSCs) and extracellular matrix, it is not surprising that Ad vectors replicating in neoplastic cells will likely fail to eradicate this aggressive tumor type. Because the TGFβ receptor (TGFBR) is expressed on both neoplastic cells and hPSCs we inserted the TGFBR targeting peptide CKS17 into the hypervariable region 5 (HVR5) of the capsid protein hexon with the aim to generate a replicating Ad vector with improved activity in complex tumors. We demonstrated increased transduction of both pancreatic cancer cell lines and of hPSCs and enhanced cytotoxicity in co-cultures of both cell types. Surface plasmon resonance analysis demonstrated decreased binding of coagulation factor X to CKS17-modified Ad particles and *in vivo* biodistribution studies performed in mice indicated decreased transduction of hepatocytes. Thus, to increase activity of replicating Ad vectors we propose to relax tumor cell selectivity by genetic hexon-mediated targeting to the TGFBR (or other receptors present on both neoplastic and non-neoplastic cells within the tumor) to enable replication also in the stromal cell compartment of tumors, while abolishing hepatocyte transduction, and thereby increasing safety.

## Introduction

Pancreatic carcinoma belongs to the most fatal human malignancies in the western countries having the lowest survival rate of any cancer [1,2]. The reasons are rapid tumor growth, early emergence of metastases, and diagnosis at an advanced stage. To date, the response to current standard therapies (surgery, radio- and chemotherapy) is limited. Thus, other strategies are urgently needed and gene therapy approaches with viral vectors might represent a new avenue for pancreatic cancer patients. Adenoviral (Ad) vectors have been widely used in clinical cancer therapy studies. Despite of promising preclinical data Ad vectors used in the treatment of pancreatic cancers have revealed only poor clinical efficacy [3,4]. Barriers explaining these disappointing results include i) the strong liver tropism of human Adenovirus type 5 (HAdV-5; short: Ad5), ii) the complex morphology of pancreatic cancers and the low expression of the primary Ad receptor on tumor cells, and iii) insufficient intratumoral spreading of non-replicating or conditionally-replicating vectors.

Because of the rapid progression and early onset of metastases of pancreatic ductal adenocarcinomas (PDACs) intravenous administration of Ad vectors would be required to reach disseminated metastases. During vascular transport, however, Ad5 interacts with a variety of circulating soluble factors such as coagulation blood factors [5–7], natural antibodies, and complement [8] resulting in a strong uptake by different liver cell types, e.g. hepatocytes, liver macrophages (Kupffer cells) [9,10], and liver sinusoidal endothelial cells (LSECs) [11,12]. Although the serial binding of Ad5 to its primary receptor CAR [13] and αvβ3 and αvβ5 integrins [14] is critical for cell entry *in vitro*, these interactions do not appear to be required for hepatocyte transduction, at least in mice [15]. Instead, several groups have identified a different Ad uptake mechanism which relies on blood coagulation factors [5,6,16], in particular factor X (FX), in mediating hepatocyte transduction [5,7]. FX binds via its Gla domain to different hypervariable regions (HVR) of the hexon capsomers [7,16] and interacts with membrane-localized heparan sulfate proteoglycan (HSPGs) [6], thereby “bridging” the virus to the hepatocyte surface. Recently, another function of FX binding to Ad particles has been described, showing that FX protects Ad particles from attack by the classical complement pathway, allowing liver transduction [17]. In addition to FX, another uptake mechanism has been identified. Several groups have demonstrated uptake and clearance of Ad particles from blood by Kupffer cells (and LSEC) by scavenger receptors, natural antibodies and complement [8,18,12].

Pancreatic carcinomas—like other carcinomas—have a complex tissue composition. Besides neoplastic cells, stromal components are found within the tumor encompassing non-neoplastic cell types including stromal cells, endothelial cells and macrophages, and extracellular matrix (ECM) components (e.g. collagens, fibronectins). In many cases, cancer cells account for a minor contribution to the cellular mass within the tumor. Frequently, the stromal cells and the ECM completely enclose tumor cell nests. This tight physical barrier formed by the stroma might prevent current Ad vectors (targeted to tumor cells only) to transduce adjacent tumor cell areas and to spread throughout the tumor. Stromal cells of pancreatic carcinomas—designated as activated pancreatic stellate cells (PSCs) – play a central role in tumor growth and desmoplasia. In mice, coinjection of pancreatic cancer cells and human PSCs (hPSCs) has been shown to result in an accelerated tumor growth highlighting the importance of hPSCs in pancreatic cancer [19,20].

A complex mutual interaction between cancer cells and non-neoplastic PSCs is orchestrated through secretion of different growth factors including transforming growth factor beta (TGFβ). Accordingly, most pancreatic cancer cell lines express normal or even high levels of type II TGFβ receptor (TGFBRII) [21–23], and analysis of PDACs revealed increased TGFBRII expression compared to normal pancreas tissue [24,25]. Because of the low CAR expression (Ad5 receptor) on pancreatic carcinomas or other malignancies [26–28], TGFBRII might be a candidate receptor target to overcome limited tumor transduction by Ad vectors.

For complete tumor destruction efficient spreading of Ad5 vectors within the tumor tissue is needed. In principle, intratumoral spreading can be achieved by using replicating (oncolytic) Ad vectors. Compared to replication-deficient, E1-deleted (ΔE1) Ad vectors, oncolytic vectors can replicate and destroy tumor cells by releasing progeny virions which are able to infect neighbouring cells until—ideally—the whole tumor is destroyed. Due to safety considerations, the replication of oncolytic Ad vectors, in general, is usually restricted to cancer cells, either by using tissue-/tumor-specific promoters to control E1A expression, or by introducing mutations into E1A and/or E1B, the latter in principle disabling viral replication in non-neoplastic cell types. Considering the complex composition of pancreatic tumors (as described above), such vector design, however, will unlikely result in the eradication of carcinomas.

Towards our aim to generate an oncolytic Ad5 vector retargeted from hepatocytes to disseminated tumor tissues, we replaced the hypervariable region 5 (HVR5) of hexon (involved in FX binding and hepatocyte transduction) by the synthetic targeting peptide CKS17, which is homologous to a conserved domain found in retroviral envelope proteins [29–31]. Of interest for targeting both pancreatic carcinoma cells and PSCs, the heptadecapeptide CKS17 contains a putative TGFβ active-site motif [32] that mediates binding to TGFBRII which is upregulated in the majority of pancreatic carcinomas. Indeed, we found that CKS17-modified Ad vectors could employ a TGFBRII-dependent cell entry mechanism to transduce CAR-negative pancreatic cancer cells and primary hPSCs, resulting in an increased cytolytic efficacy *in vitro*. Moreover, replacement of hexon HVR5 by CKS17 reduced binding to FX and led to decreased vector uptake in hepatocytes *in vivo* in mice.

Taken together, these results indicated that Ad5 vectors with reduced hepatocyte tropism and increased targeting to different cell types within the tumor—in particular cancer and stromal cells—might overcome some of the main barriers (significant hepatocyte transduction, inefficient transduction of target cells and limited intratumoral spreading due to the complex tumor structure) for efficient tumor targeting and destruction of pancreatic cancers.

## Material and Methods

### Cell lines

N52.E6 cells are based on human amniocytes stably transformed by E1A and E1B of Ad5) [33] and were cultivated in α-MEM medium (Gibco, Life Technologies, Darmstadt, Germany) supplemented with 10% fetal calf serum (FCS) and 2 mM glutamine (Glutamax; Gibco). The A549 cell line is a human lung adenocarcinoma epithelial cell line that was obtained from the American Type Culture Collection (ATCC No. CCL-243). A549 cells were maintained in MEM medium (Gibco) supplemented with 10% FCS and 2 mM glutamine. Established human pancreatic tumor cell lines Panc1 (ATCC No. CRL-1469), and MiaPaCa (ATCC No. 1420), and the early human pancreatic tumor cell line UlaPaCa [34] were cultivated in DMEM/Ham´s F12 media (PAA, GE Healthcare, Coelbe, Germany) supplemented with 10% FCS and 2 mM glutamine. Primary human pancreatic stellate cells (hPSC), isolated as previously described [19,35], were kept in DMEM/Ham´s F12 media supplemented with 20% FCS and 2 mM glutamine. The Chinese hamster ovary K1 (CHOK1, ATCC No. CCL-61) cell line lacking the coxsackie and adenovirus receptor (CAR) was grown in DMEM medium supplemented with 10% FCS and 2 mM glutamine. The murine macrophage cell line Raw 264.7 (ATCC No. CRL-2278) was cultivated in RMPI-1640 medium (Gibco) supplemented with 10% FCS and 2 mM glutamine. Cell lines were grown under standard conditions at 37°C, 95% humidity and 5% CO_2_.

### Viruses and adenoviral vectors

All vectors were derived from HAdV-5 (short: Ad5). Ad1stGFP is an ΔE1 Ad5 vector described previously [36]. AdGFPhCKS17 and AdGFPhWt are ΔE1/E3 Ad vectors. All three vectors express GFP under the control of an hCMV immediate early promoter in place of the E1 region. In addition, AdGFPhCKS17 has been hexon modified by replacing 13 amino acids of the hypervariable region 5 (HVR5) corresponding to Ad5 sequences nucleotide (nt.) 19,645 to 19,684 (the numbering is according to the HAdV-5 sequence from GenBank accession number AC_000008) with the synthetic retroviral CKS17 peptide [37] flanked by additional amino acids serving as spacer for better peptide display [38]. The encoding sequence of the CKS17 peptide used in this work is flanked by short sequences encoding the spacer region (small letters) and adjacent Ad5 sequences (underlined): CAAGTGGAAATGCAATTTTTCTCGgggTTACAGAATCGTAGAGGCCTAGATCTACTATTCCTAAAAGAGGGAGGTTTGctgggcgggCCTAAGGTGGTATTGTACAGT. For vector production all ΔE1 and ΔE1/E3 Ad vectors were produced in N52.E6 cells.

The replication–competent vector AdhCKS17 (being wildtype for Ad5 E1) carries the same hexon-modification as its non-replicating counterpart AdGFPhCKS17. AdhCKS17 and the hexon-unmodified control vector (AdhWt) were generated by inserting the Ad5 E1 region and for AdhCKS17 the CKS17 peptide encoding nucleotide sequence into the bacmid pBelo-pGS66 based on pGS66 [33] by homologous recombination (Gene Bridges, Heidelberg, Germany), followed by vector regeneration from bacmid. Human Ad5 wild-type virus (Ad5Wt; kindly provided by Albert Heim, Hannover Medical School, Hannover, Germany), AdhWt and AdhCKS17 were propagated in A549 cells.

All vectors were purified as previously described [39] by a CsCl density step gradient followed by a continuous CsCl density gradient. The vectors were desalted by a PD-10 size exclusion column (GE Healthcare, Dassel, Germany) and were stored at -80°C in PBS supplemented with 10% (v/v) glycerol. After vector purification the vector DNA was isolated from vector particles by the QiaAmp DNA Mini Kit (Qiagen, Hilden, Germany) as described by the manufacturer`s protocol and verified by restriction analysis and partial sequencing. Infectious and particle vector titers were determined by DNA slot blot analysis [40] using a Ad5 fiber-specific probe encompassing nt. 31,042 to 32,390 of the Ad5 sequence. The inverse bioactivity was calculated by dividing the number of physical by the number of infectious particles.

### Ad vector mediated transduction *in vitro*

2x10^4^ hPSCs or 2x10^5^ tumor cells were seeded in 24 well plates. About 16 hours later cells were transduced with ΔE1 GFP-expressing Ad5 vectors (Ad1stGFP, AdGFPhCKS17 or AdGFPhWt) at indicated multiplicity of infection (MOI) of physical particles (pMOI) or infectious particles (iMOI) per cell.

For the evaluation of vector-mediated GFP expression cells were detached from culture plates 24 hours after transduction using a prewarmed trypsin solution (Gibco). After the addition of PBS containing 20 mM EDTA and 10% (v/v) FCS (to inactivate trypsin) and centrifugation at 300 x g for 5 minutes, cells were resuspended in FACS buffer containing 2% (v/v) FCS, 20 mM EDTA in PBS. Cells were assessed by flow cytometric analysis using a Becton-Dickinson FACSCalibur without gating (Becton-Dickinson, Franklin Lakes, NJ, United States of America). Relative transduction units refer to the percentage of GFP-positive cells or the mean fluorescence of all cells. To determine the relative Ad genome content within cells, cells were washed twice with prewarmed PBS for 5 minutes 2 hours after transduction and were then detached with 200 μl of 50 mM EDTA in PBS. After addition of 200 μl 0.8 N NaOH for cell lysis, cell lysates were subjected to DNA slot blot analysis [40]. Relative Ad vector genome contents expressed as relative transduction units were calculated and set to 100% for A549 cells transduced with a hexon-unmodified Ad vector.

### AdWt replication *in vitro*

1x10^6^ tumor cells per 6 cm dish seeded the day before were infected with Ad5Wt at a slot blot adjusted MOI of 1. Two and 48 hours post infection cells were washed with prewarmed PBS and detached by trypsination. After inactivating trypsin by FCS cells were pelleted (300 x g, 5 min, 4°C) and washed with 1 ml of PBS. After another centrifugation step (300 x g, 5 min, 4°C) each cell pellet was resuspended in 200 μl PBS and total DNA was isolated with the QiaAmp DNA Mini Kit (Qiagen, Hilden, Germany) according to the manufacturer’s protocol. Two micrograms of DNA were subjected to slot blot analysis using an Ad5 fiber-specific probe. Ad replication was calculated from the ratio of Ad genome contents obtained at 48 and 2 hours post infection and set to 100% for Ad5Wt-infected A549 cells.

### Production of infectious Ad particles *in vitro*

1x10^6^ tumor cells per 6 cm dish seeded the day before were infected with Ad5Wt at a slot blot adjusted MOI of 1. Forty-eight hours post infection cells were washed with prewarmed PBS and detached by trypsination. After inactivating trypsin by FCS cells were pelleted (300 x g, 5 min, 4°C) and washed with 1 ml of PBS. After another centrifugation step (300 x g, 5 min, 4°C) each cell pellet was again resuspended in 1 ml PBS. Cells were then disrupted by repeated freezing and thawing, and lysates were cleared by centrifugation (1800 x g, 5 min, 4°C). To remove unpacked adenoviral genomes, cellular DNA and RNA, lysates were treated with 5 units of Benzonase endonuclease (Sigma-Aldrich, Taufkirchen, Germany) and incubated at 37°C for 30 min. Two and 10 microliters of the lysate were used to reinfect 2x10^5^ A549 cells seeded the day before. Two hours after reinfection, A549 cells were washed twice with prewarmed PBS. Then, cells were detached with 50 mM EDTA in PBS and lysed with 0.4 N NaOH. The number of infectious particles was determined by DNA slot blot analysis [40] using a Ad5 fiber-specific probe and the number of infectious particles per tumor cell was calculated.

### CAR expression on pancreatic cells

5x10^4^ hPSCs and 5x10^5^ tumor cells seeded the day before were washed with PBS and detached with trypsin. After trypsin inactivation with FCS, cells were pelleted (300 x g, 5 min, 4°C) and washed with 1 ml of washing buffer (ice-cold PBS containing 5% (v/v) FCS). After centrifugation the supernatant was aspirated, anti-CAR primary antibody (1 μg per sample, RmcB clone; Millipore, Schwalbach, Germany) was added and incubated for 30 minutes on ice. Cells were washed again and incubated with the secondary antibody, Alexa 488, F(ab´)_2_ fragment (1 μg per sample; Invitrogen, Darmstadt, Germany), for 30 minutes on ice. As a control served cells, which were stained with the secondary antibody only. After another washing step cells were resuspended in washing buffer and subjected to flow cytometric analysis to determine CAR expression calculated from the mean fluorescence intensity of all cells.

### Steady-state levels of TGFBRII on pancreatic cells

Pancreatic hPSCs and tumor cells were lysed in NP40 lysis buffer (50 mM Tris/ HCl, pH 8.0, 150 mM NaCl, 5 mM EDTA, 0.15% (w/v) Nonidet P-40) in the presence of complete protease inhibitor cocktail (Roche, Penzberg, Germany) and 1 mM PMSF for 1 hour on ice. After repeated freezing and thawing cell debris was pelleted (21,000 x g, 15 min, 4°C). The protein concentration of the lysate supernatant was determined (Bio-Rad protein assay; Biorad, Munich, Germany). Fifty μg of the lysates were analyzed by 8% SDS-PAGE and immunoblotting. TGFBRII was detected by an anti-TGFBRII-specific polyclonal antibody from rabbit (sc-1700; Santa Cruz Biotechnology, Heidelberg, Germany). The mouse monoclonal antibody DM1A from Sigma-Adrich (Taufkirchen, Germany) was used for the detection of α-Tubulin serving as a loading control.

### Competitive virus transduction assays

To demonstrate an altered cell entry pathway of a CKS17 hexon-modified Ad vector, monolayers of A549 cells were pretreated with soluble fiber knob (kindly provided by Pierre Boulanger, Université Lyon, Lyon, France) at 1,000 fold molar excess over fiber protein or with a polyclonal anti-TGFBRII antibody from rabbit (sc-1700; Santa Cruz Biotechnology, Heidelberg, Germany) at 1,000 fold molar excess over hexon protein for 30 min at room temperature. As an isotype control for the anti-TGFBRII antibody the same volume of rabbit serum was used. AdGFPhCKS17 and the control vector were added at a pMOI of 100 without removing the competitiors. After incubation for 1.5 hours incubation at room temperature cells were washed twice with culture medium to remove remaining virus and competitors. Thereafter, cells were cultured at 37°C for additional 2 hours to determine Ad genome levels from isolated total DNA by qPCR or for 24 hours to assess GFP expression (expressed as the mean fluorescence of all) by flow cytometry.

### Release of progeny virions

1x10^6^ hPSCs or 2.5x10^6^ A549, Panc1 or UlaPaCa cells seeded the day before were washed once with PBS. After the addition of medium and replicating Ad vectors (AdhWt or AdhCKS17) at an infectious MOI of 20, cells were incubated for 6 hours at 37°C. Thereafter, the medium containing Ad vectors was removed and the cells were washed twice before fresh medium was added to the cells. Samples of the medium were taken at 48 and 72 hours post infection, centrifuged to remove cells, mixed with glycerol (to obtain a 10% final concentration of glycerol), and stored at -80°C. At 72 hours post infection, cells were scraped off in the remaining medium and also stored at -80°C.

For subsequent quantitative PCR analysis of released Ad particles in the medium DNA was isolated from the medium samples taken 48 and 72 hours post infection using GenElute™ Mammalian Genomic DNA Miniprep kit (Sigma-Aldrich, Taufkirchen, Germany). Quantitative PCR analysis was performed by amplification of the Ad5 E4 gene using the Stratagene 2× Brilliant II SYBR Green QRT-PCR Master Mix Kit, 1-Step in a Stratagene 3005P qPCR machine. To generate a standard curve, medium was spiked with 1x10^8^ particles of AdhWt and serially diluted. Oligonucleotides: E4-sense (5′- tagacgatccctactgtacg -3′), E4-antisense (5′- ggaaatatgactacgtccgg -3′). Forty cycles with the following thermal protocol were performed: melting (95°C; 30 seconds), annealing (60°C; 30 seconds), and elongation (72°C; 30 seconds). For analysis, the number of E4 copies was determined by E4 Ct (dR) values and the standard curve and referred to the cell numbers seeded.

The number of infectious particles released into the medium at 48 and 72 hours post infection and within the cells and the medium collected at 72 hours was determined by plaque assay. Cells collected together with medium were disrupted by repeated freezing and thawing as described above. To remove cell debris, samples were centrifuged at 400 x g for 10 minutes, and supernatants were transferred to fresh tubes. The medium taken at the indicated time points was directly used in this assay. Serial dilutions of the supernatants and the medium were used to infect A549 cells that were seeded at a number of 7.5x10^5^ cells the day before. A 5% (w/v) PeqGold low-melting agarose solution (dissolved in PBS and autoclaved) was 1:4 diluted with medium containing 2% serum and kept at 37°C. After 2 hours the virus-containing medium was carefully aspirated and the cells were overlaid with prewarmed (37°C) 1.25% (w/v) agarose solution. After 15 minutes at room temperature to allow the agarose to solidify, the cells were incubated at 37°C. The number of plaques was counted 10 days after infection.

### Cell viability assay

The cytolytic activity of the hexon-modified vector was analyzed in both single and co-cultures of cancer cells and hPSCs. Thus, 2 x10^3^ cells in single cell cultures in 96-well plates per well and each 1 x10^3^ of tumor cells and hPSCs in co-cultures seeded the day before were transduced with AdhCKS17 and the AdhWt control, respectively, at different particle MOIs ranging from 1 to 1,000. Seven days after infection, cell viability was determined according to the manufacturer´s protocol using the Cell-TiterGlo system (Promega, Mannheim, Germany).

### SPR analysis

To analyse the interactions of the hexon-modified (AdGFPhCKS17) and the hexon-unmodified control (AdGFPhWt) vectors with factor X (FX) surface plasmon resonance (SPR) experiments were carried out using a Reichert SR7500DC SPR instrument (Reichert Technologies, Buffalo, New York, United States of America). Human coagulation FX (Haematologic Technologies, Essex Junction, Vermont, United States of America) was covalently immobilized onto one flow cell of a (Carboxymethyldextran hydrogel biosensor chip (CMD500m chip purchased from XanTec bioanalytics GmbH, Duesseldorf, Germany)) by amine coupling according to the manufacturer’s instructions. Standard amine coupling in absence of any protein (dummy coupling) was performed on a second flow cell yielding a reference flow cell. Signals obtained for the FX-surface were subtracted by signals obtained for the reference flow cell according to standard procedure. Only reference subtracted sensorgrams are shown. Removal of glycerol from virus stock solutions and exchange of buffer to 10 mM HEPES pH 7.4, 150 mM NaCl, 5 mM CaCl_2_, and 0.005% Tween 20 was achieved by gel filtration using PD MiniTrap G-25 columns (GE Healthcare, Dassel, Germany). Vectors serially diluted with the same buffer to obtain concentrations ranging from 1.25x10^8^ to 1x10^9^ physical particles per millilitre were passed over the chip for 3 min at a flow rate of 25 μl/min. The dissociation phase consisted of buffer flow at 25 μl/min for 5 min and was followed by a regeneration step with regeneration buffer (10 mM HEPES (pH 7.4), 150 mM NaCl, 3 mM EDTA, and 0.005% Tween 20), which was injected for 50 s at a flow rate of 25 μl/min.

### FX-dependent transduction

To investigate the influence of hexon modification on interaction with FX the FX-dependent transduction rates of CKS17 hexon-modified Ad vector were analyzed. Therefore, 2x10^4^ A549 cells were intensively washed twice with PBS one day after seeding. Then the cells received either serum-free medium (control) or serum-free medium containing 8 μg/ml FX (physiological concentration). Ad vectors with a particle MOI (pMOI) of 1,000 were added to the cells. Cells were then incubated for 3 hours at 37°C and washed three times with PBS. After the addition of serum-containing cell culture medium, cells were incubated at 37°C for 24 hours. Then, cells were harvested as described above and the GFP expression was analyzed by flow cytometry analysis.

### *In vivo* biodistribution

To investigate the influence of reduced FX binding of hexon-modified Ad vector AdGFPhCKS17 *in vivo*, a biodistribution study in mice was performed. Female BALB/c mice (6 to 8 weeks of age) were purchased from Charles River, Sulzfeld, Germany. All animal experiments were approved by the Animal Care Commission of the Government of Baden-Wurttemberg (Permit Number: 975) and were in strict accordance with institutional guidelines. Clodronate was a gift from Roche Diagnostics GmbH (Mannheim, Germany). It was encapsulated in lipsosomes as described earlier [41]. For Kupffer cell depletion, 200 μl of clodronate liposomes was injected into the tail vein. After 24 hours, Ad vector particles (3 x10^10^) were injected intravenously into the tail vein of mice in a total volume of 200 μl (in HEPES-buffered saline). Forty-five minutes or 72 hours after injection the mice were anaesthetized by isoflurane (Forene; Abbott, Ludwigshafen, Germany) inhalation, livers were perfused with PBS, and organs were collected. Thereafter, mice were sacrificed by bilateral thoracotomy. Then, the organs were snap-frozen in liquid nitrogen and stored at -80°C for subsequent DNA isolation (qPCR analysis) or homogenization (fluorimetric analysis).

### Quantitative PCR analyses

Quantitative PCR analysis was performed by amplification of the Ad5 fiber gene using the Stratagene 2× Brilliant II SYBR Green QRT-PCR Master Mix Kit, 1-Step in a Stratagene 3005P qPCR machine. To normalize for the cellular DNA content murine or human β-actin was used. To generate standard curves, liver DNA of naive mice (biodistribution study) or genomic DNA isolated from human A549 cells (competition experiment) was spiked with pGS66 containing the Ad5 fiber gene. Oligonucleotides: murine β-actin-sense (5′-GCTGTGTTCTTGCACTCCTTG-3′), murine β-actin-antisense (5′-CGCACGATTTCCCTCTCAGC-3′), human β-actin-sense (5′-GCTCCTCCTGAGCGCAAG-3′), human β-actin-antisense (5′-CATCTGCTGGAAGGTGGACA-3′), fiber-sense (5′-GCTACAGTTTCAGTTTTGGCTG-3′), fiber-antisense (5′-GTTGTGGCCAGACCAGTCCC-3′). Forty cycles with the following thermal protocol were performed: melting (95°C; 30 seconds), annealing (60°C; 30 seconds), and elongation (72°C; 30 seconds). For analysis, the fiber Ct (dR) values were normalized to β-actin Ct (dR) values of the same sample.

### Fluorimetric analysis of mouse liver homogenates

Five hundred micrograms of perfused and snap-frozen liver were homogenized in 1ml homogenization buffer (50 mM Tris/HCl, pH 7.4, 150 mM NaCl, 1 mM EDTA, 1% (v/v) NP-40, 0.25% (w/v) sodium desoxycholate) with a conical tissue grinder (Wheaton, Millville, NJ, USA), transferred into a 1.5 ml reaction tube and incubated for 10 minutes at room temperature. The samples were centrifuged for 10 minutes, 20,000 x g, 4°C. The supernatant clear fraction was transferred into a new reaction tube, centrifuged for 10 minutes, 20,000g, 4°C, and diluted 1:500 to 1:20,000 in homogenization buffer. The GFP fluorescence was analyzed in a fluorescence spectrometer LS50B (Perkin Elmer, Waltham, MA, United States of America) at 488 nm excitation wavelength and 512 nm emission wavelength. Relative units were calculated and the arbitrary units for the unmodified vector were set to 1.

### Neutralization of Ad vectors by natural IgM

The influence of hexon modification on recognition by natural IgM antibodies was investigated in a neutralization assay. 2x10^4^ A549 seeded the day before were washed once with PBS, and serum-free medium was added. 1x10^7^ Ad particles which had been incubated for 20 minutes at 37°C with 30 μl of hirudinized (Refludan®, Celgene, Munich, Germany) plasma from NMR1 mice or with serum-free medium (control) in a total volume of 32 μl, were added to the cells. After 3 hours of incubation at 37°C cells were washed once with PBS, and after addition of serum-containing medium the cells were incubated at 37°C for 24 hours, followed by flow cytometry to determine GFP-expression.

### Uptake of Ad vectors by murine macrophages

1x10^5^ Raw 264.7 cells seeded the day before were washed once with PBS, and serum-free medium was added.

2x10^8^ Ad vector particle which had been incubated with 30 μl of hirudinized plasma from NMR1 mice or with serum-free medium (control) (in a total volume of 35 μl) for 20 minutes at 37°C were added to the cells. After 45 minutes of incubation at 37°C cells were washed twice with PBS, and total DNA was isolated according to the manufacturer´s protocol. Quantitative PCR analysis of Ad particles taken up by murine macrophages was performed by amplification of the Ad5 E4 gene using the Stratagene 2× Brilliant II SYBR Green QRT-PCR Master Mix Kit, 1-Step in a Stratagene 3005P qPCR machine. To generate a standard curve, genomic DNA of Raw 267.4 cells spiked with 1x10^5^ to 1x10^8^ particles of AdhWt was isolated and also subjected to PCR analysis. Primer for PCR: E4-sense (5′-tagacgatccctactgtacg-3′), E4-antisense (5′-ggaaatatgactacgtccgg-3′). Forty cycles with the following thermal protocol were performed: melting (95°C; 30 seconds), annealing (60°C; 30 seconds), and elongation (72°C; 30 seconds). For analysis, the number of E4 copies was determined by E4 Ct (dR) values and the standard curve.

### Statistics

In all figures, means ± standard deviations are shown. If not indicated differently, the significance of the data was determined by using the unpaired Student’s t-test (2-sided) or Welch´s test on normal distributions. In case of non-normal distributions, experiments were analyzed by Mann-Withney-Wilcoxon test.

## Results

### Inefficient Ad transduction of pancreatic cells

**Fig 1:**
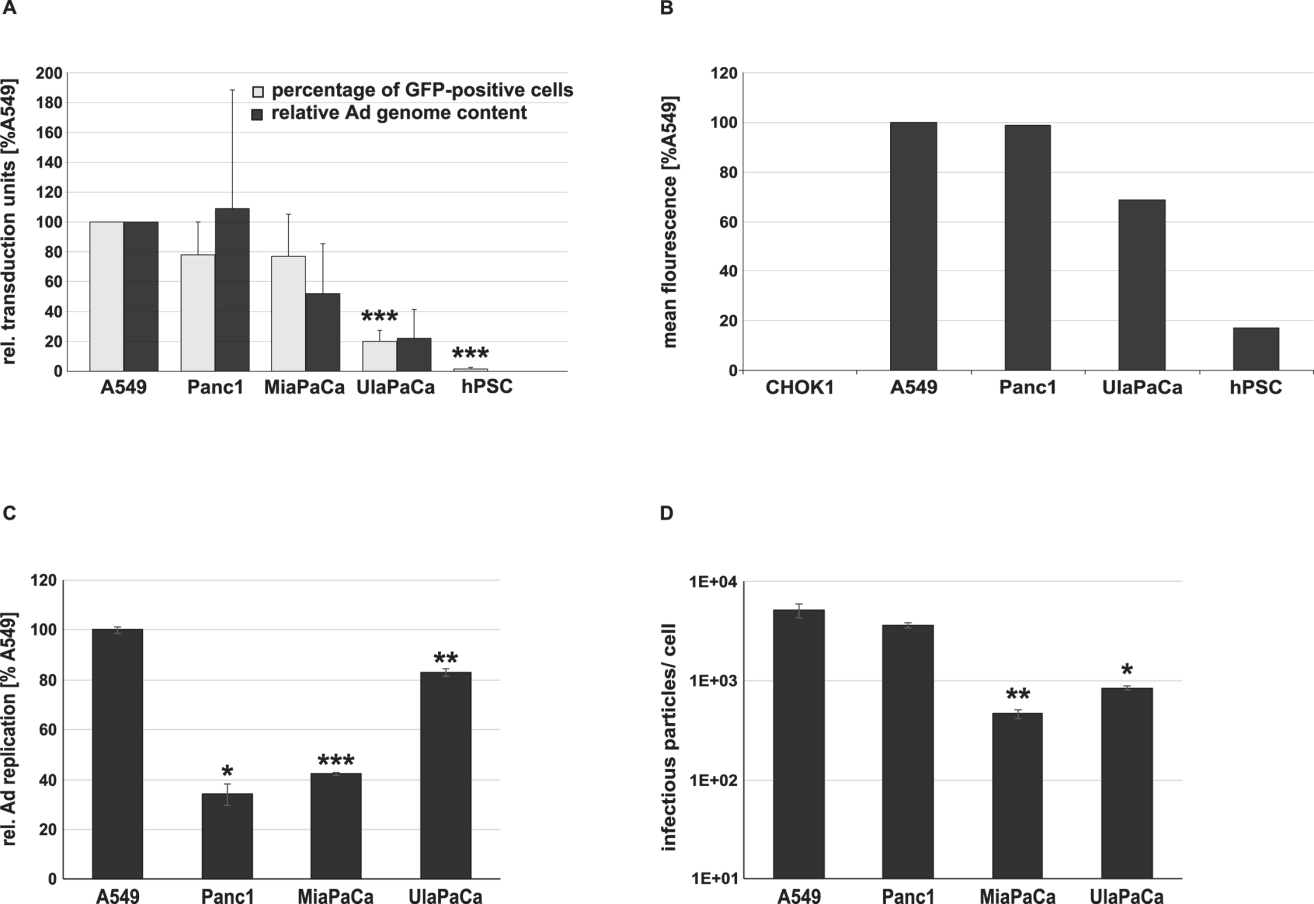
Limitations of Ad vector-mediated transduction of early passage pancreatic cancer cell lines and primary hPSCs. (**A**) 2x10^4^ hPSCs or 2x10^5^ tumor cells were transduced with ΔE1 Ad1stGFP at a MOI of 5. To determine the Ad transduction rate the cells were subjected both to flow cytometry for detection of GFP expression (24 h.p.i.) expressed as the percentage of GFP-positive cells and to slot blot analysis to determine their relative Ad genome content (2 h.p.i.). (**B**) CAR levels on different cell lines and hPSCs were determined by flow cytometry as detailed in the Materials and Methods section. To analyze Ad replication rates and production of progeny virions in different cells, respectively, wildtype Ad5 (Ad5Wt) was used for infection of different cell lines at a slot blot adjusted actual MOI of 1. (**C**) Cells were harvested 2 and 48 hours p.i., genomic DNA was isolated and subjected to slot blot analysis using an Ad5 fiber-specific probe. Ad replication is expressed as the ratio of Ad genomes 48 h.p.i./ 2 h.p.i. in comparison to A549 cells (set to 100). (**D**) Forty-eight hours after infection cells harvested, lysed by repeated freezing and thawing, and treated with Benzonase. Two and 10 microliters of the lysate were used to reinfect A549 cells. The number of infectious particles was determined by DNA slot blot analysis [40] using a Ad5 fiber-specific probe and the number of infectious particles per tumor cell was calculated. * P < 0.05, ** P < 0.01 *** P < 0.005, n = 2–4.

In this study we evaluated the usage of Ad5 vectors to target both neoplastic and non-neoplastic cell types found in pancreatic cancers. Possible barriers for an efficient Ad infection can occur at different levels including virus attachment and uptake, transgene expression and replication. To investigate vector attachment and uptake we first assessed susceptibility of different human pancreatic cancer cell lines and hPSCs to Ad vector transduction *in vitro*. Compared to A549 control cells and the established pancreatic cancer cells (Panc1 and MiaPaCa), the early cancer cell line UlaPaCa exhibited a markedly reduced transduction efficacy with only 20% of the cells transduced compared to A549 cells (Fig. 1A). Primary hPSCs (passage 6 to 8) exhibited almost no GFP expression and appeared to be resistant to Ad vector transduction. To exclude differences due to cell type-specific variations of promoter activity, the relative amounts of intracellular Ad genomes after transduction were determined. Results obtained (Fig. 1A, black columns) were in accordance with those obtained from flow cytometry analyses suggesting that efficiency of Ad vector transduction of early tumor cells and hPSCs was very low.

### Low steady state levels of CAR receptor in early pancreatic cancer and hPSC cells

The coxsackie and adenovirus receptor (CAR) is expressed on many cell types and, at least *in vitro*, it serves as the main receptor for primary cellular uptake of most human adenovirus types, including Ad5. In contrast to established Panc1 cells, CAR expression levels of early pancreatic tumor cells were slightly (UlaPaCa) or strongly (hPSCs) reduced compared to A549 cells indicating that low CAR expression probably contributed to poor transduction of neoplastic and stromal cells by Ad vectors (Fig. 1B).

### No limitation of Ad replication in early pancreatic cancer and hPSC cells

Another limitation for spreading of replicating Ad vectors in pancreatic cancer could be a block in genome replication within cancer cells. Therefore, we analyzed, if and to what extent replication of Ad5 and production of progeny virus in pancreatic cancer cells was blocked. Replication of the vector genomes (Fig. 1C) and virus production (Fig. 1D) varied among the pancreatic cancer cells and was only slightly reduced in some of the cell lines compared to A549 cells. Since hPSCs were resistant to vector transduction at low MOI, replication of the viral genome in hPSCs was not investigated. Together these data pointed to a transduction defect of early pancreatic cancer cells and stromal cells rather than to limited viral replication as a reason for limited spreading.

### Hexon modification enhances transduction of early pancreatic cancer cells

**Fig 2:**
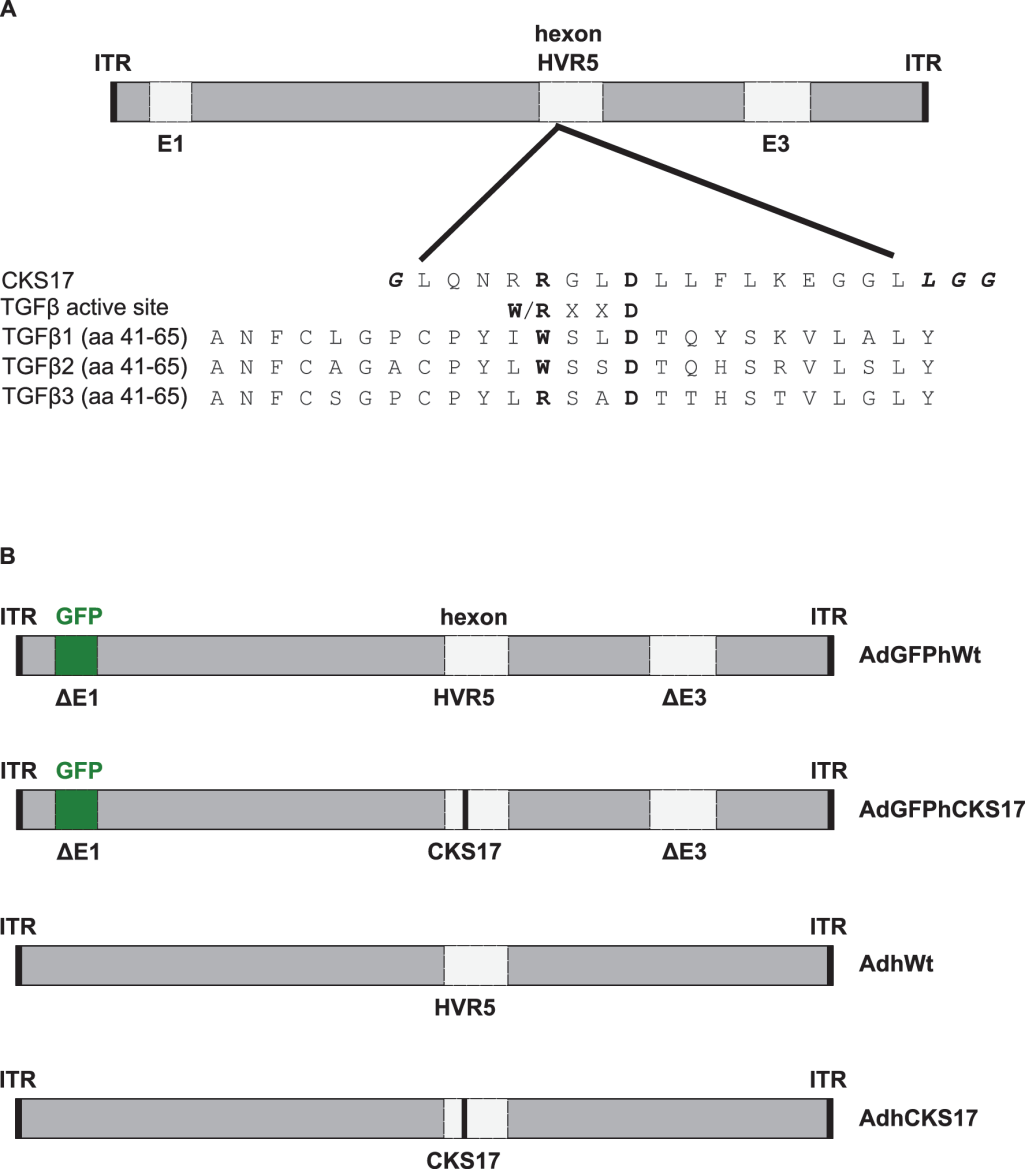
Hexon modification and vectors used in this study. (**A**) Modification of HVR5 of Ad5 hexon. Amino acids 269 to 281 of HVR5 were replaced by a synthetic CKS17 peptide, which contains the TGFβ active site motif (bold letters) and is flanked by two different short linker sequences (bold italic letters). (**B**) Schematic presentation of ΔE1/E3 first-generation and replicating Ad vectors harbouring the unmodified or the CKS17-modified hexon protein.

**Fig 3:**
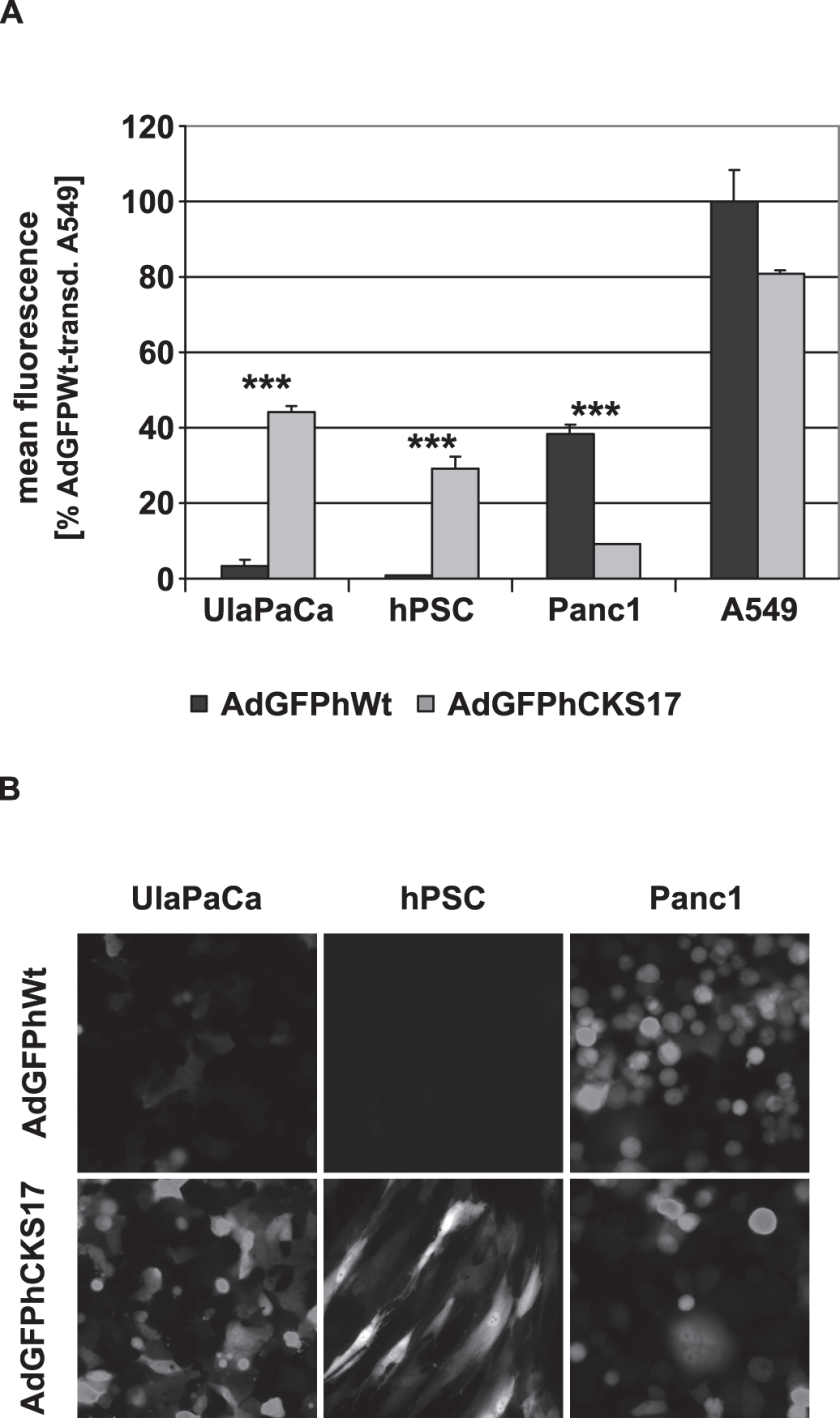
Transduction of cancer cell lines and primary hPSCs with hexon-modified vector. 2x10^5^ tumor cells or 2x10^4^ hPSCs, respectively, were transduced with AdGFPhCKS17 and control vector AdGFPhWt at a particle MOI of 1,000. Twenty-four hours after infection cells were (**A**) subjected to flow cytometry to determine the mean fluorescence of all cells or (**B**) analysed by microscopy (100 x magnification). *** *P* < 0.005, n = 3.

To overcome the restriction in Ad transduction of early pancreatic cancer cells and of primary hPSC cells, a previously described synthetic peptide designated CKS17 [32] was used as a targeting ligand and introduced in place of the HVR5 of hexon (Fig. 2). Like the TGFβ ligands, the CKS17 peptide harbours an R/WXXD motif which mediates binding to the TGFBR [37]. First, we analyzed the influence of the CKS17 hexon modification on transduction efficiency. Transduction of the established Panc1 cell line with AdGFPhCKS17 resulted in a reduction of GFP expression compared to vector with unmodified hexon (Fig. 3A and 3B). In contrast, both the early pancreatic cancer cells UlaPaCa and primary hPSCs displayed a more than 10-fold and 35-fold enhanced transduction efficacy, respectively. These results revealed that the limited transduction of both relevant cell types by Ad vectors was overcome by the CKS17-hexon modification.

### CKS17 hexon-modified Ad vectors employ an alternative, TGFBRII-mediated cell entry mechanism

**Fig 4:**
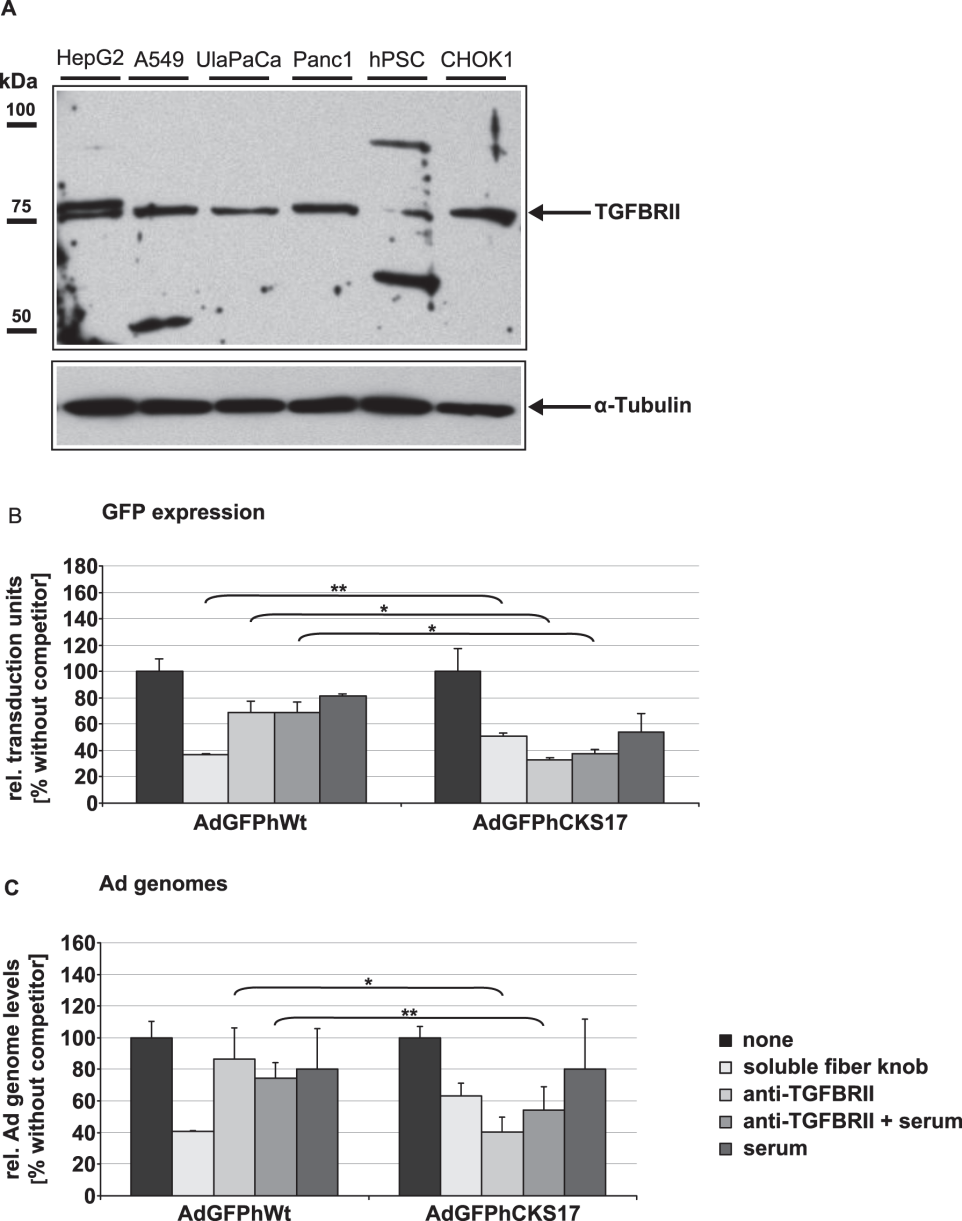
Cellular uptake inhibition experiments with soluble fiber knob protein and TGFBRII-specific antibody. (**A**) Lysates from pancreatic hPSCs and tumor cells were analyzed by SDS-PAGE and immunoblotting. TGFBRII was detected by an anti-TGFBRII-specific polyclonal antibody from rabbit (sc-1700). A549 cells were pre-incubated with soluble fiber knob protein, TGFBRII-specific antibody or rabbit serum (serving as isotype control), respectively, and transduced with the replication-deficient vectors AdGFPhCKS17 and AdGFPhWt (control) at a particle MOI of 100. After two hours the medium was replaced with fresh medium and incubated for additional 2 and 24 hours to (**B**) analyze GFP expression by flow cytometry or to (**C**) determine relative Ad genome levels by qPCR using total DNA isolated from cells. * *P* < 0.05, ** *P* < 0.01, n = 3.

Prior to the evaluation of TGFPBRII tropism of the CKS17 hexon-modified vector we performed Western blot analysis of cell lysates from pancreatic cancer cells and hPSCs and detected the presence of TGFBRII (Fig. 4A), a receptor overexpressed in PDACs [24,25]. In subsequent transduction competition experiments using the CKS17 vector we could show that the interaction of fiber with CAR leads to Ad attachment followed by transduction, which can be efficiently blocked by soluble fiber knob protein. Compared to the control vector, AdGFPhCKS17 exhibited a reduced transduction in the presence of soluble fiber knob (Fig. 4B and 4C). The remaining transduction by the control vector might be explained by receptor-independent fluid phase pinocytosis, an entry mechanism demonstrated with fluorescent Ads in A549 cells [42]. Next we tested the effect of the CKS17 peptide (when presented on the capsid protein) on Ad uptake mechanism. Preincubation with a TGFBRII-specific antibody (Fig. 4B and 4C) significantly blocked transduction by CKS17 hexon-modified vector suggesting a TGFBRII-mediated cell entry mechanism.

### Hexon modification results in an enhanced oncolytic activity of Ad vectors

**Fig 5:**
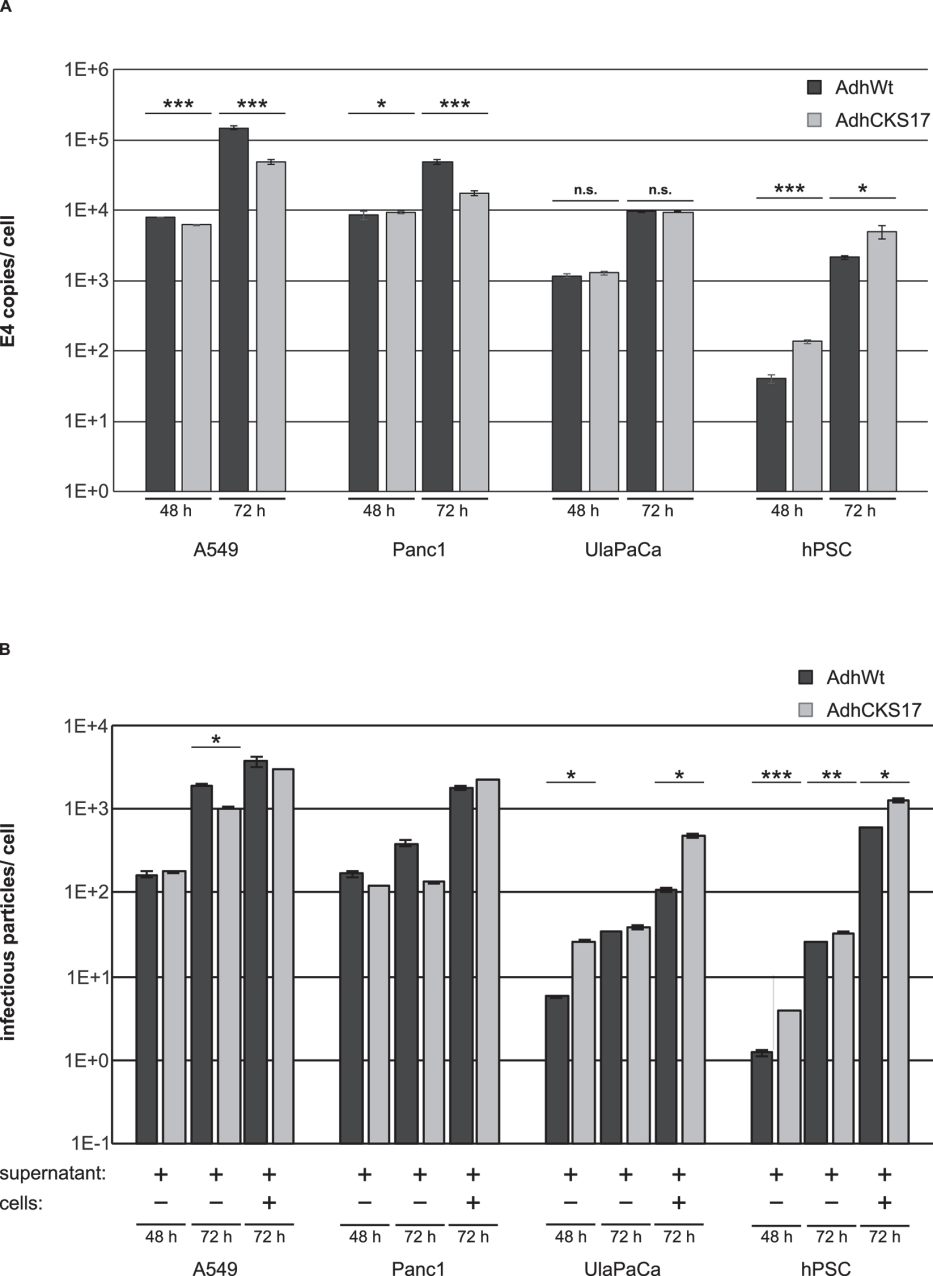
DNA replication and virus release of hexon-modified Ad vector in pancreatic cells. 1 x 10^6^ hPSCs or 2.5x10^6^ A549, Panc1 or UlaPaCa cells were seeded. On the next day, replicating Ad vectors (AdhWt or AdhCKS17) were added at an infectious MOI of 20. Six hours post infection cells were washed with PBS to remove unbound virus, and fresh medium was added to the cells. The supernatants and the cell lysates were collected during the course of infection (at 48 and 72 hours post infection). (**A**) To determine the number of physical particles found in the supernatant at the indicated time points, viral DNA was isolated and subjected to quantitative PCR to analyse the Ad genome number. (**B**) The numbers of infectious particles per cell (viral release) within the supernatants or the cell lysates (indicated by “-”or “+”) were assessed by plaque assay. * *P* < 0.05, ** *P* < 0.01, n = 2.

For successful tumor treatment efficient virus replication (besides enhanced Ad transduction) is required for virus spreading and subsequent tumor growth inhibition and destruction. Therefore, a replicating Ad vector carrying the same modification in the hexon protein (AdhCKS17) and a control vector (AdhWt) were generated. To determine the production of progeny virions during the course of infection—critical for vector spreading within solid tumors—the number of physical and infectious particles released into the cell culture medium and within the cells at 72 h was determined (Fig. 5A and 5B). Although the numbers of physical particles released from A549 and Panc1 cells into the supernatant were significantly lower for AdhCKS17 than for AdhWt, the number of infectious particles from both vectors was similar in these cells. By contrast, UlaPaCa showed no difference regarding physical particles, but significantly increased infectious particle numbers for AdhCKS17. Finally, hPSC cells infected with the hexon-modified vector showed elevated numbers of both physical and infectious particles compared to hPSC cells infected with the wild-type virus control.

**Fig 6:**
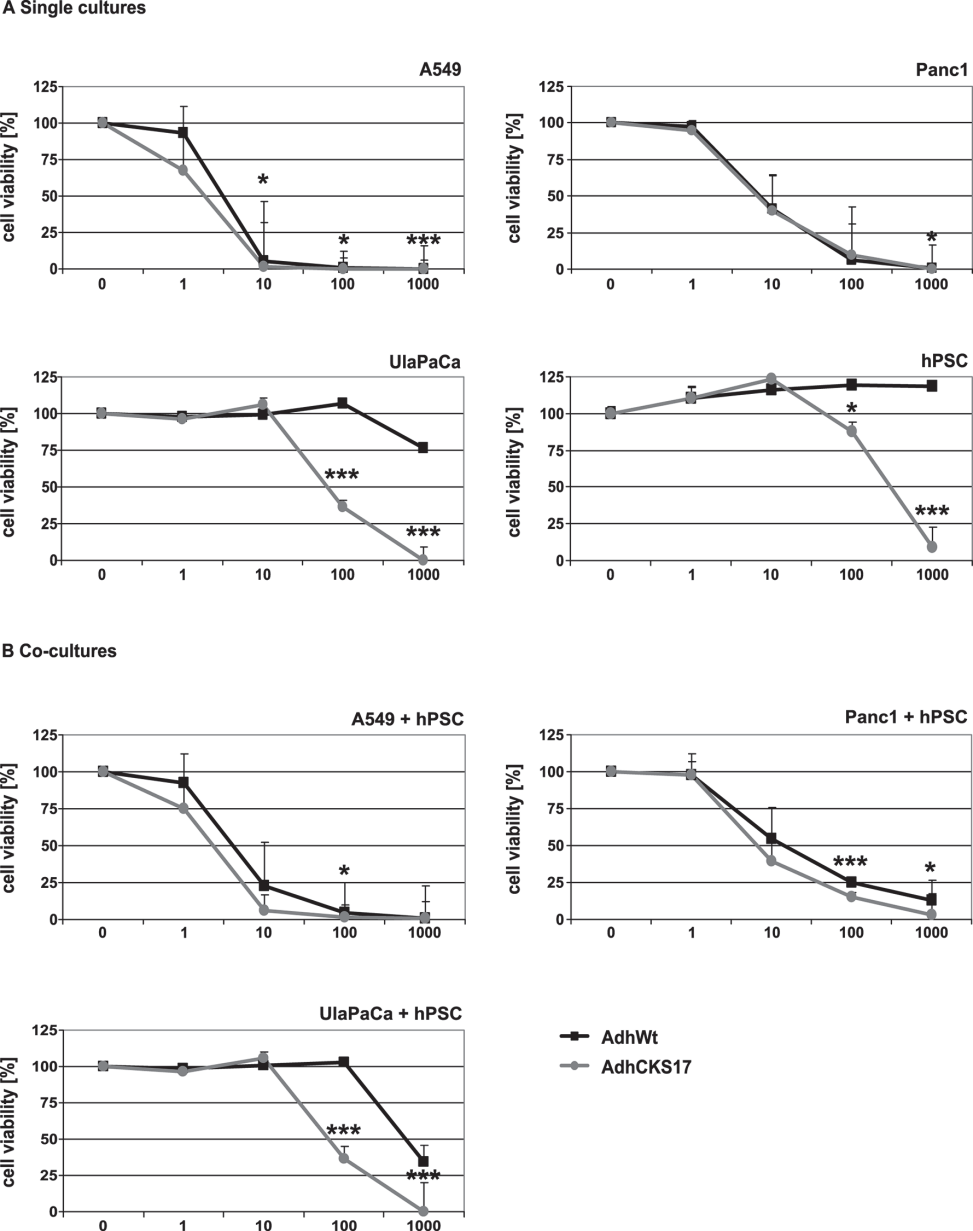
Cytolytic activity of replicating Ad vectos in single cell culture or co-cultures. (**A**) 2x10^3^ cells for single cell cultures or (**B**) 1x10^3^ of both tumor cells and hPSC in co-culture experiments were infected with the replication competent vectors AdhCKS17 and AdhWt (control) at different particle MOIs. After seven days the cells were lysed and cell viability was determined with the CellTitre Glo system. * *P* < 0.05, *** *P* < 0.005, n = 3.

Subsequent *in vitro* studies evaluated the cytolytic potential of the hexon-modified Ad vector. Single cell cultures of the early passage pancreatic cells (UlaPaCa and hPSC) infected with AdhCKS17 had a significantly reduced cell viability (Fig. 6A) compared to those infected with the unmodified control vector. Considering the complex architecture of pancreatic tumors, the cytolytic potential of Ad vectors was also investigated in co-cultures of cancer cells and hPSC (Fig. 6B). All co-cultures exhibited significantly decreased cell viability after infection with the hexon-modified Ad vector compared to the wild-type virus control. The result from UlaPaCa/hPSC co-cultures indicated that the CKS17 hexon modification improved virus spreading and cytolysis of two cell types found in pancreatic tumors.

### Hexon modification with the CKS17 peptide affects binding to coagulation factor X

**Fig 7:**
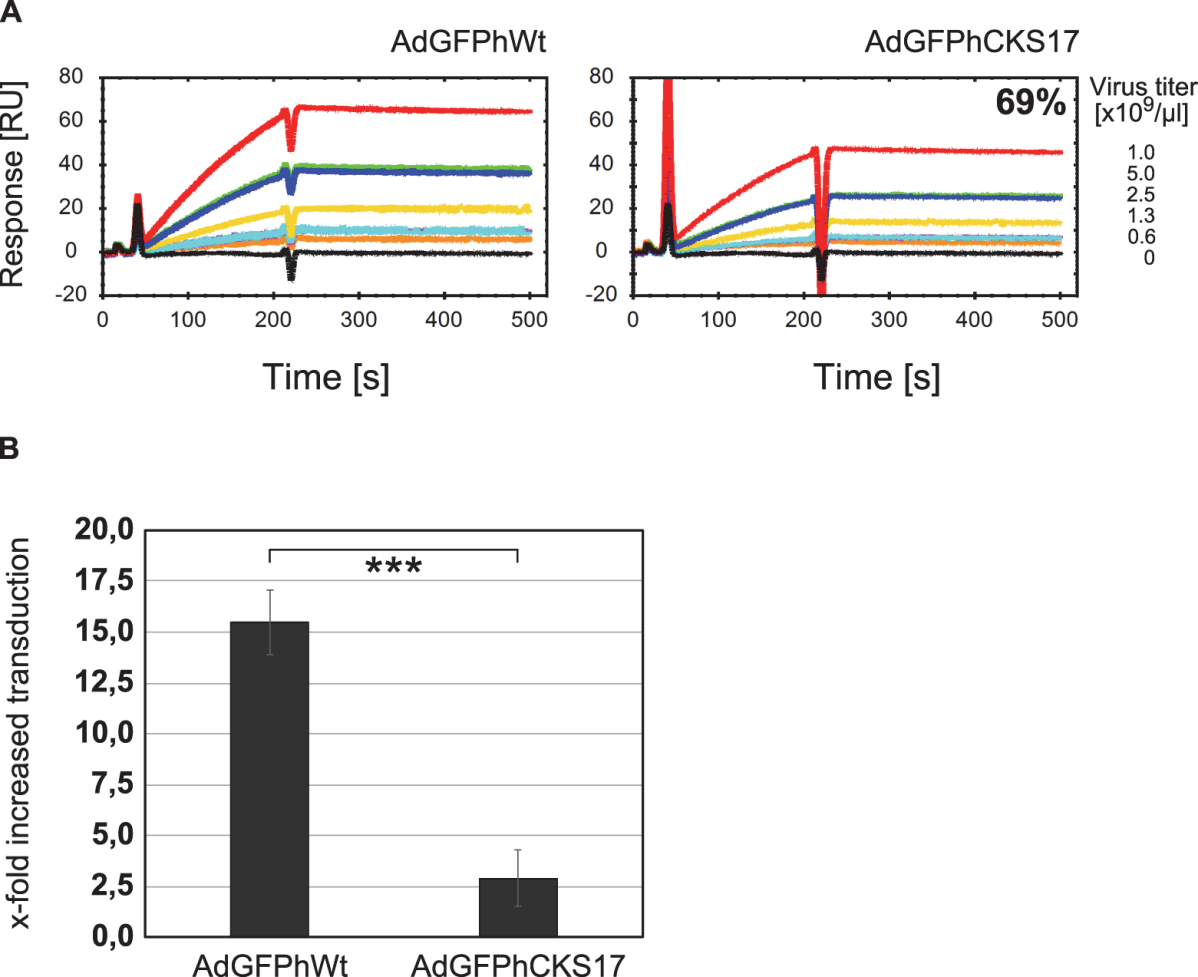
Analysis of FX binding. (**A**) To analyze the interactions of the hexon-modified (AdGFPhCKS17) and the hexon-unmodified control (AdGFPhWt) vectors with factor X (FX) surface plasmon resonance (SPR) experiments were carried out. Human coagulation FX was covalently immobilized onto one flow cell of a Carboxymethyldextran hydrogel biosensor chip (CMD500m chip) by amine coupling. A reference surface was blank immobilised. Several vector dilutions (ranging from 0 to 1x10^9^ particles/μl) diluted with Ca^2+^-containing buffer were passed over both chip surfaces. Reference surface subtracted sensorgrams are shown. Sensor chip surfaces were regenerated between vector injections by injection of an EDTA-containing buffer, which disrupts the Ca2^+^-dependent viron:FX interaction. Each concentration series contained two concentrations which were injected in duplicate (as indicated) to probe reproducibility. (**B**) To investigate the influence of the reduced FX binding on Ad transduction rates, 2x10^4^ A549 cells seeded the day before were transduced with AdGFPhWt or AdGFPhCKS17 (pMOI of 1,000) in the absence (PBS) or presence of FX (8 μg/ml, physiological concentration). After 24 hours cells were harvested and the GFP expression was analyzed by flow cytometry analysis. *** *P* < 0.005, n = 3 (analyzed by Mann-Withney-Wilcoxon test).

It has been shown that FX binds to different HVRs of the hexon protein [7,16,43]. Here, we tested to which extent the replacement of the HVR5 by CKS17 influenced FX binding (Fig. 7A). Using surface plasmon resonance (SPR), we confirmed direct and Ca^2+^-dependent binding to Ad virus particles to immobilized FX. For the CKS17 hexon-modified vector 30% reduced binding to FX was detected. The reduction of FX binding was associated with a decrease in FX-dependent cell transduction (Fig. 7B). This is in accordance with previous results [44,45]. Thus, the CKS17 modification reduced but did not completely abolish FX binding.

### Altered liver tropism of CKS17-hexon modified Ad vectors

**Fig 8:**
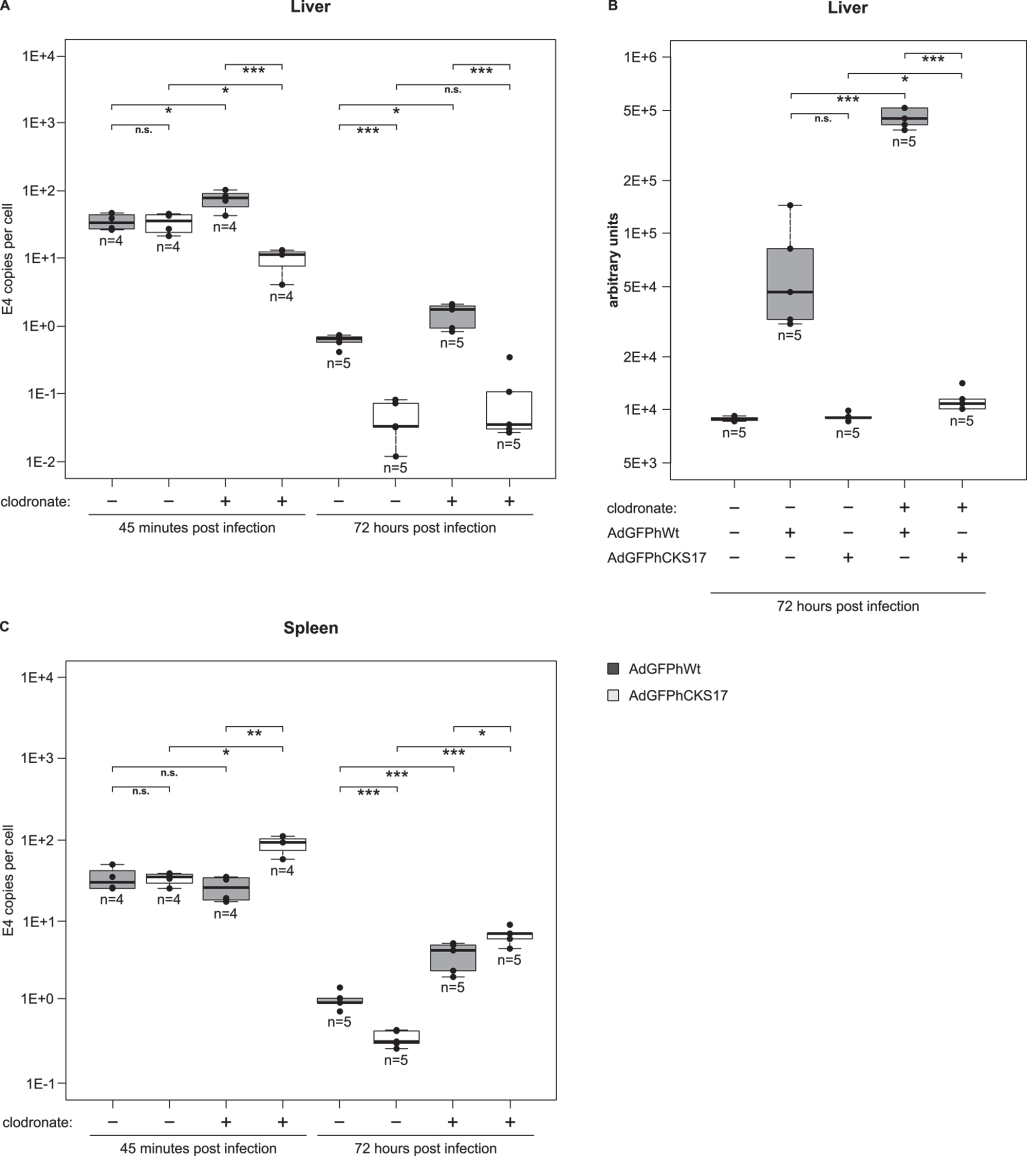
Influence of hexon modification on biodistribution. For Kupffer cell depletion, 200 μl of clodronate liposomes were injected into the tail vein of BALB/c mice. After 24 hours, 3x10^10^ viral particles of AdGFPhWt or AdGFPhCKS17 were injected intravenously into the tail vein of mice (n = 4 or 5), and organs were collected 45 minutes or 72 hours later. (**A**) Relative Ad genome levels were determined from total DNA isolated from liver obtained 45 minutes or 72 hours after infection. (**B**) GFP expression was measured in liver lysates obtained 72 hours after infection by fluorimetry. (**C**) Analysis of relative Ad genome levels from total DNA isolated from spleen obtained 45 minutes or 72 hours after infection. * *P* < 0.05, ** *P* < 0.01, *** *P* < 0.005, n = 4–5.

Upon systemic administration Ad5-based vectors exhibit a strong liver tropism (uptake by and activation of Kupffer cells and hepatocyte transduction) regulated and mediated by FX. In light of the reduced, but not abolished, FX binding of the CKS17 vector we analyzed the role of hexon modification on liver uptake and biodistribution in mice (Fig. 8). At an early time point (45 min postinjection) Ad genome levels in liver and spleen of AdGFPhWt or AdGFPhCKS17 injected mice were almost identical (Fig. 8A and 8C). At a late time point (72 h postinjection) the overall Ad genome levels in these organs were substantially decreased with AdGFPhCKS17 DNA being significantly reduced compared to those of the unmodified vector (Fig. 8A and 8C). In Kupffer cell-depleted animals, the CKS17 vector—compared to the control vector—showed reduced Ad genome levels in liver, but increased levels in spleen both at 45 minutes and at 72 h postinfection (Fig. 8A and 8C). In liver, GFP expression after injection of AdGFPhCKS17 was significantly reduced compared to the control independent of clodronate treatment (Fig. 8B).

### Effect of hexon modification on IgM-mediated vector neutralization and macrophage uptake

**Fig 9:**
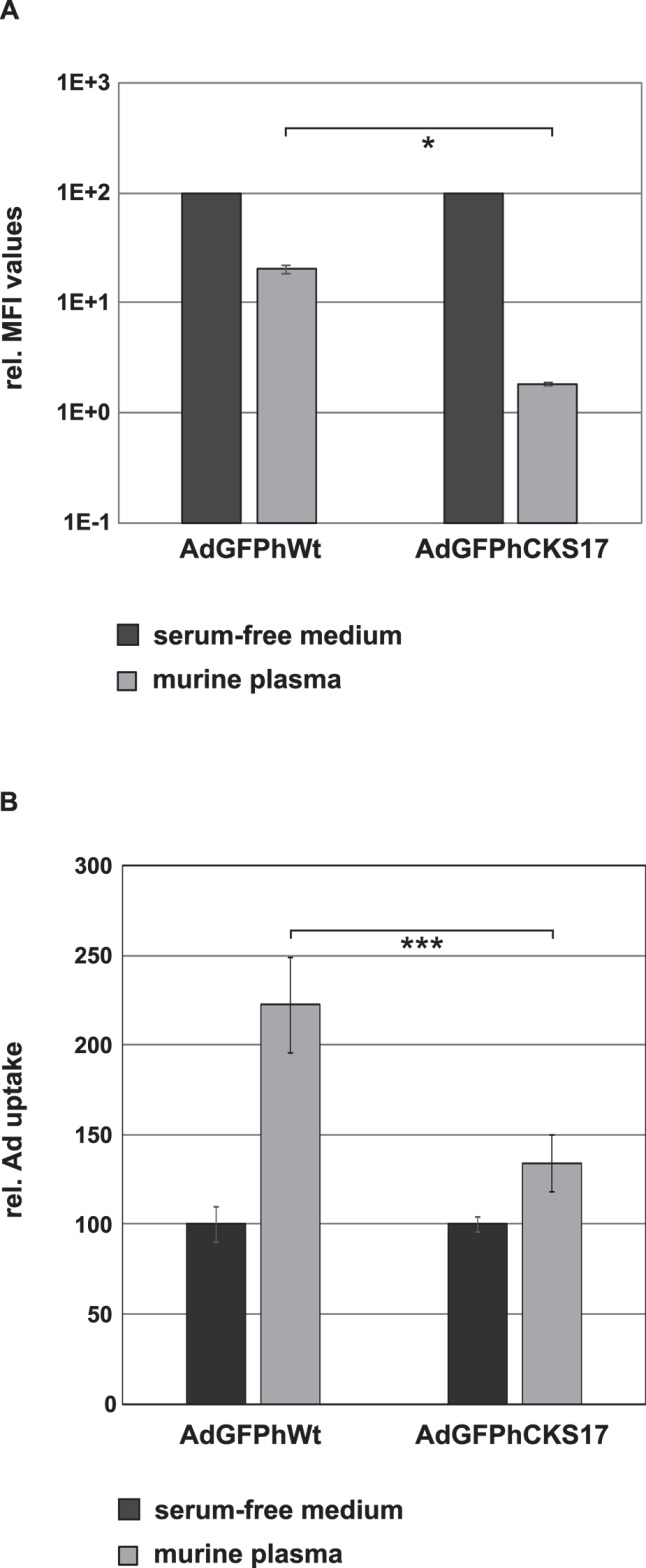
Influence of murine plasma on neutralisation and macrophage uptake of the CKS17 vector. (**A**) The influence of hexon modification on binding to natural IgM antibodies was investigated in a neutralisation assay. There, 2x10^4^ A549 cells seeded the day before were transduced with AdGFPhCKS17 or AdGFPhWt (control) pMOI of 500 in the absence (serum-free medium) or presence of murine plasma and subjected to flow cytometry analysis to determine the GFP-expression 24 hours later. (**B**) 1 x 10^5^ Raw 264.7 cells were seeded. Sixteen hours later the cells were transduced with AdGFPhWt or AdGFPhCKS17 at a physical MOI of 2,000 pre-incubated with serum-free medium (control) or murine plasma. After 45 minutes of incubation at 37°C, total DNA of the cells was isolated and subjected to quantitative PCR analysis.

Natural IgM antibodies are part of the innate immune defense system and recognize antigens in the blood even in the absence of prior antigen exposure. They have a relatively low affinity for monomeric antigens [46] but have a high avidity due to multivalent binding to repetitive structures (e.g. hexon within the capsid). Xu and colleagues have shown that recognition of Ad particles by natural IgMs [17] is inhibited by FX binding to hexon [17]. We first examined the ability of natural IgM antibodies to bind and neutralize hexon-modified Ad particles *in vitro*. Using a neutralization assay and serum from naïve mice, we found that CKS17-modified Ad particles were neutralized 10-fold more efficiently than the unmodified vector (Fig. 9A). As natural antibodies are involved in Ad clearance by Kupffer cells *in vivo* [8], we investigated the uptake of Ad particles by murine macrophages in the presence of (naïve mouse serum containing natural IgMs). This exposure resulted in a 1.6 fold decreased uptake of hexon-modified Ad vector in macrophages compared to unmodified control vector (Fig. 9B).

## Discussion

Patients suffering from pancreatic cancer have a very low less than 9% [2] 5-year survival rate resulting from late diagnosis, infiltrating and rapid tumor growth, high malignancy and resistance to standard therapies. In PDACs, the stroma including the ECM-producing myofibroblast-like cell type PSC [35] and ECM components frequently constitutes the majority of the tumor mass and forms a physical barrier around tumor cells. In healthy pancreas of humans, hPSCs comprise about 4% of all pancreatic cells, whereas in PDACs a marked increase in the number of activated hPSCs has been shown [47,48].

In general, Ad vectors used for tumor treatment are targeted to cancer cells, disregarding the presence of non-neoplastic cell types such as hPSCs, the latter playing a central role in tumor growth and desmoplasia in PDAC [19,20].

In patients with pancreatic cancer treatment results with conditionally replicating Ad vectors (CRAds) have been disappointing due to no, or low, expression of the primary Ad attachment receptor CAR on tumor cells leading to poor transduction [26–28]. Accordingly, our results from infection experiments in early passage normal and neoplastic cells of the pancreas (hPSC and UlaPaCa) showed that Ad5 transduction (Fig. 1A), but not replication (Fig. 1C), was the limiting factor for cell killing. To improve transduction several strategies have been pursued. Hexon has been modified i) to avoid binding of pre-existing neutralizing anti-Ad antibodies, ii) to target receptors other than CAR, and iii) to alter the known liver tropism. Examples for genetic modification have been the replacement of HVRs from Ad5 by corresponding sequences from other Ad serotypes [44], the insertion of targeting peptides [45,49], or the introduction of point mutations in HVR5 or HVR7 to ablate FX binding [43]. Although modification of hexon HVRs by inserting high-affinity binding targeting peptides has been proven difficult probably due to altered virus trafficking as a result of impaired endosomal escape [50], several groups have shown that HVR5 can be used as a peptide insertion platform [38,44]. So far, mostly non-replicating Ad vectors have been hexon modified, although hexon-modified oncolytic Ad vectors have shown promising results leading to an increased survival of tumor-bearing mice [51,52].

TGFBRII is expressed on pancreatic tumor cell lines and pancreatic cancers [21–25] and is associated with decreased survival [53]. To target TGFBRII, we inserted the TGFBRII-binding peptide CKS17 [37] into the HVR5 of hexon (Fig. 2A). Transduction experiments indicated a substantial increase in transduction of the early pancreatic tumor cell line UlaPaCa and primary hPCS by the CKS17 vector (Fig. 3), hPSCs being highly resistant to unmodified Ad vector. The lack of transduction of hPSCs by wildtype Ad vector is contrary to a previous publication by Brock *et al*., 2006, where PSCs from rat have been transduced quite efficiently by Ad5 [54]. In fact, when comparing Ad vector-mediated transduction rates of human versus rat PSCs, we observed in rat cells an almost 5-fold increased transduction compared to human PSCs (data not shown). The observed low transduction of Panc1 cells by CKS17 vector is not due to a lack of binding receptors, since Panc1 expresses similar levels of CAR and TGFBRII as A549 cells (Fig. 1B and Fig. 4A). Previously, it has been suggested for other hexon peptide insertions that these negatively affected postbinding steps [50]. As transduction rates of CKS17 and control vector were similar in A549 cells, a severe defect in the postbinding steps, such as particle disassembly, in CKS17-modified virions is unlikely. Additionally, production levels were modestly reduced in Panc1 cells, compared to control particles (Fig. 5B). These data indicated that the CKS17 modified vector was suitable to specifically target early pancreatic cancer and stroma cells.

Competition experiments (Fig. 4B and 4C) showed that the CKS17 vector was blocked by a TGFBRII-specific antibody as well by soluble fiber knob, suggesting that the CKS17 hexon modified Ad vector employed both CAR- and TGFBRII-dependent pathway for cell entry. While this set of experiments delivered valuable hints for TGFBRII targeting, it must be noted, however, that so far there is no direct proof of TGFBRII being the target receptor. Nevertheless, CKS17 hexon modification appears to be a rational choice for targeting Ad vectors to both tumor cells and hPSCs to overcome limited Ad transduction.

For efficient virus spreading within tumor tissue efficient virus replication and release are required. The CKS17 hexon modification, shown to mediate increased transduction in UlaPaCa and hPSCs, resulted in an enhanced production of infectious progeny virions in these cell types. Moreover, infection of UlaPaCa/hPSC co-cultures by the replicating CKS17 vector (AdhCKS17) showed an enhanced cytolytic activity compared to the control vector (Fig. 6), suggesting that the CKS17 hexon modification might improve spreading in complex tumors with neoplastic and non-neoplastic cell types.

The liver tropism of Ad5 vectors after systemic administration depends on interactions with different blood components such as blood coagulation factors, antibodies, and complement [8,55,56]. These interactions mediate the sequestration of Ad5 to the liver, where resident liver macrophages (Kupffer cells) can deplete more than 90% of an injected vector dose [9,57]. Liver sinusoidal cells (LSECs) also contribute to virus uptake in the liver [11]. Thus, for hepatocyte transduction, Ad5 must circumvent uptake by Kupffer cells or LSECs and pass liver fenestrae [58–60] to enter the space of Disse, where hepatocytes are localized. FX is regarded as a bridging factor between Ad5 hexon and cellular heparan sulfate *in vitro* [6], thus promoting liver transduction *in vivo* [5,7,16]. Binding of FX to Ad5 is mediated by different HVRs of the hexon protein [6,43,44]. Insertion of the CKS17 sequence in the FX-binding site of hexon HVR5 [43,44] resulted in a ~30% reduced binding of hexon by FX compared to unmodified vector as shown by SPR analysis (Fig. 7A). Previously, mutations within HVR5 and HVR7 led to a 10% to almost 100% reduction of FX binding [43]. Subsequent transduction experiments showed that the CKS17-modified vector is impaired in its ability to use FX for cell transduction (Fig. 7B). These findings indicated that insertion of CKS17 into HVR5 of hexon reduced but did not completely abolish binding to FX.

Several groups have previously shown that HVR5 hexon-modified and FX-binding ablated Ad vectors exhibit an altered liver tropism *in vivo* [7,16,44]. At early time points (~ one hour after infection) the total amount of Ad found in the liver was independent of FX as shown in FX-depleted mice [16,61,62] or with FX-binding ablated vectors [7,16,43,44]. In the absence of FX (binding), however, an increased uptake of Ad by Kupffer cells mediated by natural antibodies and complement was observed [8,17] contributing to a rapid clearance of Ad from the liver and a low hepatocyte transduction. This is in agreement with our results showing that the CKS17 modification did not affect liver targeting at an early time point after injection, but led to significantly reduced genome levels and expression at 72 hours after injection (Fig. 8A and 8B). Interestingly, in the absence of Kupffer cells, Ad genome levels of the CKS17 vector were reduced at an early time point suggesting that in the presence of Kupffer cells an increased number of CKS17 virions was retained within the liver by this cell type. In a murine macrophage cell line, the CKS17 vector showed an increased uptake in the presence of murine plasma (Fig. 9B) that—according to unpublished data from our group (Krutzke *et al*.) – depended on natural antibodies and complement. Thus, a reduction of FX binding sensitizes the CKS17 vector to natural antibodies and complement (Fig. 9A), as it has been previously shown for wild-type Ad particles with normal FX binding *in vivo*. These findings suggest that in the absence of FX binding CKS17 vector particles become more efficiently opsonized and are taken up by Kupffer cells probably via receptors recognizing complement or antibodies [8,63,64]. This protein decoration of the vector contributes to sequestration of Ad particles by these cells, which otherwise seems to be mainly mediated by scavenger receptors [8,65].

Although our data indicated that the CKS17 peptide insertion into HVR5 reduced hepatocyte transduction *in vivo* while increasing uptake into early pancreatic cancer cells and hPSCs *in vitro*, it is presently unclear whether this will translate to an enhanced anti-tumor activity *in vivo*. Complementary strategies to further improve bioavailability could be used such as site-specific genetic-chemical modification (using polymers such as polyethylene glycol or N-(2-hydroxypropyl)methacrylamide) [18,66–68] or engineering of capsid proteins to further reduce unwanted interactions with non-cellular or cellular blood components or with the reticulo-endothelial system (Kupffer cells, LSECs) [55].

With the strategy presented here we address three barriers for Ad-mediated therapy of PDAC: inefficient transduction of early pancreatic cancer cells, limited vector spreading in complex tumors, and unwanted vector uptake in hepatocytes. We showed that the CKS17-hexon modification resulted in improved transduction of an early neoplastic cancer cell line and also of non-neoplastic hPSCs, likely via targeting of TGFBRII, and in improved cell killing in mixed cell cultures. The specific position of the peptide insertion reduced, but did not abolish, FX binding. However, transduction of hepatocytes after i.v. injection was strongly reduced. *In vivo* studies will be essential as the next step to analyse this vector in murine xenotransplantation models bearing mixed-cellular tumors consisting of human cancer and stromal cells.
