# Correction: Hexon Modification to Improve the Activity of Oncolytic Adenovirus Vectors against Neoplastic and Stromal Cells in Pancreatic Cancer

**DOI:** 10.1371/journal.pone.0123844

**Published:** 2015-04-08

**Authors:** The PLOS ONE Staff

There are errors in the names and affiliations for the fourth and fifth authors. The correct names and affiliations should appear as shown here:

Christoph Q. Schmidt^3^, Andreas Wortmann^4^

3 Institute of Pharmacology of Natural Products & Clinical Pharmacology, Ulm University, Ulm, Germany

4 Tierforschungszentrum, Ulm University, Ulm, Germany

The correct citation is: Lucas T, Benihoud K, Vigant F, Schmidt C, Wortmann A, Bachem MG, et al. (2015) Hexon Modification to Improve the Activity of Oncolytic Adenovirus Vectors against Neoplastic and Stromal Cells in Pancreatic Cancer. PLoS ONE 10(2): e0117254. doi:10.1371/journal.pone.0117254
